# An Intelligent Robot Detection System of Uncontrolled Radioactive Sources

**DOI:** 10.1155/2022/1806601

**Published:** 2022-09-19

**Authors:** Jianyou Zhuang, Guibing Zheng

**Affiliations:** ^1^School of Internet of Things Technology, Hangzhou Polytechnic, Hangzhou 311400, China; ^2^General Manager's Office, Shanghai Image Sound Control Technology Co., Ltd., Shanghai 200020, China

## Abstract

In recent years, radioactive sources have been widely used in various fields (e.g., nuclear industry, agriculture, medical industry, environmental protection, and scientific research) and successfully applied to develop scientific projects, such as nuclear power generation, sewage treatment, medical diagnosis, and new material development. However, radiation sources continuously got out of control and even lost. Manual search for uncontrolled radiation sources is inefficient and prone to radiation injuries. Therefore, it is practically significant to design a radiation source detection robot. Against this backdrop, this study designs an intelligent robot detection system of uncontrolled radiation sources and develops an intelligent robot for detecting and disposing of uncontrolled radiation sources. The research results help to realize the autonomous search and disposal of radiation sources.

## 1. Introduction

Since the turn of the century, the nuclear industry has developed rapidly worldwide. In August 2019, the World Nuclear Association (WNA) released the World Nuclear Industry Status Report 2019 [1], pointing out that the global nuclear power generation has grown year by year since 2012. At the end of 2018, 55 nuclear reactors were under construction. More than 30 countries that have not used nuclear power are considering building nuclear projects or introducing nuclear energy. Suffice it to say that nuclear power is booming around the world [2–4].

Apart from the nuclear power industry, radiation sources have been widely used in various fields. For example, in the industrial field, the rapid development of nuclear technology applications has become one of the driving forces for the continuous innovation and development of new technologies, new materials, new processes, and new methods. In the field of agriculture, nuclear technology has been widely used in the fields of plant radiation mutagenesis breeding, agricultural and food radiation processing, and radiation sterility of kun and has become an important science and technology for transforming, renovating traditional agriculture, and promoting agricultural modernization. In the medical field, nuclear techniques are combined with modern medical techniques to prevent, diagnose, and treat diseases. In the field of environmental protection, nuclear analysis technology has been applied to the monitoring of atmospheric pollutants and the analysis of water and various environmental samples; the use of irradiation technology to carry out purification treatment of air, wastewater, and sludge can achieve better results than traditional processing technology is more efficient, lower energy consumption, and more accurate processing power. However, radiation sources continuously got out of control and even lost. Starting from 1979, major accidents have occurred in several nuclear power plants around the world, which destroyed a number of cities. In China, an average of 30 radiation accidents happens each year [5, 6]. In most accidents, radiation sources are feasible to leak or lost, posing an imminent threat to our life and property. According to the annual report of the National Nuclear Safety Administration, the loss or theft of radioactive sources in the eight years from 2012 to 2019. A total of 44 radiation accidents occurred in eight years, and 52 radioactive sources were lost. Among these accidents, more than half of them belong to the loss or theft of radioactive sources, mainly due to the failure of proper management of radioactive sources that have not been used for a long time or that have been decommissioned. Loss of radioactive sources in different industries always threatens the safety of human life and property. For a large-scale nuclear power plant accident, the accident area will have a very high radiation intensity. Wearing radiation protection suits cannot isolate a large dose of radiation. Manual disposal will cause great harm to human beings, so human beings cannot visit the accident site for treatment and detection. When small radiation sources are lost, it is difficult to pinpoint the position of the lost radiation sources. Manual search for uncontrolled radiation sources is inefficient and faces a high health risk. Radiation can cause harm to human health, ranging from general weakness, coughing, and fever, to organ failure and even death in severe cases. Therefore, radiation source search robots are preferred for handling radiation accidents.

The above analysis shows the practical significance of designing a radiation source detection robot. Therefore, this study establishes an intelligent robot detection system of uncontrolled radiation sources and develops an intelligent robot for the autonomous search and disposal of uncontrolled radiation sources.

## 2. Composition of Detection System

The intelligent detection system of uncontrolled radiation sources, responsible for searching and locating radiation sources, consists of three parts: an airborne source finder, a 360-degree rotating support platform, and a detection-control system. The airborne source finder contains a gamma ray detector with high and low ranges, and a neutron detector, which judges whether there are neutron rays. The 360-degree rotating support platform provides support to the finder, shielding layer, and rotating control device, and deduces the possible direction of radiation sources. The detection-control system manages and controls the finder and rotating platform, transmits the dose rate and rotation angle to the control platform of the robot, and receives the operation instructions from that platform. The workflow of the intelligent detection system for uncontrolled radiation sources is divided into four steps: the first step is to conduct inspections on the ground to search for possible radioactive sources through the robot-borne detection instrument. The second step is to perform real-time location positioning after detecting the radioactive source and transmit the location information to the intelligent detection system in real time. The third step is the system's communication module in the device communicates and transmits data such as positioning position data, nuclear radiation monitoring data, and source-finding positioning calculation results. Finally, the detection system communicates with the intelligent computing system in real time. On the one hand, the monitoring results are transmitted to the intelligent computing system, and on the other hand, the instruction information of the intelligent computing system is received.

## 3. Design of Source Finder

### 3.1. Probe Selection

The airborne source finder simultaneously monitors gamma radiation and neutron radiation. For gamma radiation monitoring, the probe combines two Geiger-Mueller (GM) tubes (VacuTec, Germany), which separately measure the gamma radiation dose of low and high ranges.  Table 1 lists the performance indices of the GM tubes provided by the manufacturer.

Concerning the high-range GM tube, the manufacturer gives the peripheral dose equivalent *H* *∗* (10), while our project requires the absorption dose rate. For Co-60, the conversion coefficient for the peripheral dose equivalent *H* *∗* (10) and air kerma Ka is 1.16. Then, the measuring range (0.1 m Sv/h–20 Sv/h) of the peripheral dose equivalent of the high-range GM tube can be converted into the measuring range of air kerma: 0.09 mGy/h–17.24 Gy/h. For the calibration source with the 137Cs scale, the conversion coefficient for the peripheral dose equivalent *H* *∗* (10) and air kerma Ka is 1.2. Then, the measuring range (0.1 m Sv/h–20 Sv/h) of the peripheral dose equivalent of the high-range GM tube can be converted into the measuring range of air kerma: 0.083 mGy/h–16.67 Gy/h. From a conservative perspective, it is suitable for the 70018 high-range GM tube to have a range of 0.09 mGy/h–16.67 Gy/h.

The absorbed dose *D* is equal to the quotient of *dε* divided by *dm*, that is, the average imparted energy *dε* imparted to a medium of mass *dm* by ionizing radiation. It calculates as follows:

(1) $$\begin{array}{c} D=d\varepsilon dm. \end{array}$$

The kerma energy *Ka* is equal to the quotient of *dEtr* divided by *dm*, which is the sum of the initial kinetic energies of all charged particles released by uncharged ionized particles in a medium of mass *dm*:

(2) $$\begin{array}{c} Ka=\frac{dEtr}{dm}. \end{array}$$

The relationship between the dose equivalent *H* *∗* (10) around photons with energies ranging from 20 keV to 10 MeV and the free air kerma energy *Ka* can be estimated as follows:

(3) $$\begin{array}{c} \frac{H*\left(10\right)}{K_{a}}=\frac{X}{\left(aX^{2}+bX+c\right)}+d\cdot \;\arctan\left(gX\right). \end{array}$$

In the formula, the unit of *H* *∗* (10)/*Ka* is Sv/Gy; *X* = ln(*E*/*E*0), *E* is the photon energy (keV); *E*0 = 9.85 keV; *a* = 1.465, *b* = −4.414, *c* = 4.789, *d* = 0.7006, *g* = 0.6519; the unit of angle is the radian. The conversion factor from free air kerma energy *Ka* to ambient dose equivalent *H* *∗* (10) is shown in Table 2.

Considering the possible impacts and harms of neutrons on instruments and personnel, our project team also designed a neutron detector to check whether neutron radiation exists. The neutron probe needs to meet the following performance requirements (Table 3).

### 3.2. Determination of Shielding Layer Thickness and Probe Measuring Range

Our project requires the finder to work normally for 3 h at the dose rate of 500 Gy/h. During the project design, it is assumed that the source term is a Co-60 radiation source, and each GM tube is protected against gamma radiation by a lead shielding layer. The half-value layer (HVL) of lead against the gamma rays from the Co-60 radiation source is 12 mm.

The lead shielding layer can be divided into two parts: the lead shielding layer for the high-range tube, and that for the low-range tube. For the 70018 high-range GM tube, the thickness of the lead shielding layer is 60 mm (five HVLs). After the shielding, the detection range of the high-range tube is expanded to 2.88 mGy/h–533.33 Gy/h. The conservative measuring range is 3 mGy/h–530 Gy/h, which is better than the requirement of our project (the finder should work normally for 3 h at the dose rate of 500 Gy/h).

The 70031 low-range GM tube is also protected by a lead shielding layer against gamma radiation. The layer is 8 mm thick (2/3 of an HVL). After the shielding, the detection range of the low-range tube is expanded to 0.133 *μ*Gy/h–4 mGy/h. The conservative measuring range is 0.15 *μ*Gy/h–4 mGy/h.

When both high-range and low-range GM tubes are protected by the lead shielding layer, the detection range of the entire finder can meet the monitoring requirement for the radiation field of 0.15 *μ*Gy/h–530 Gy/h.

### 3.3. Preparation of Lead Shielding Layer

**Figure 1:**
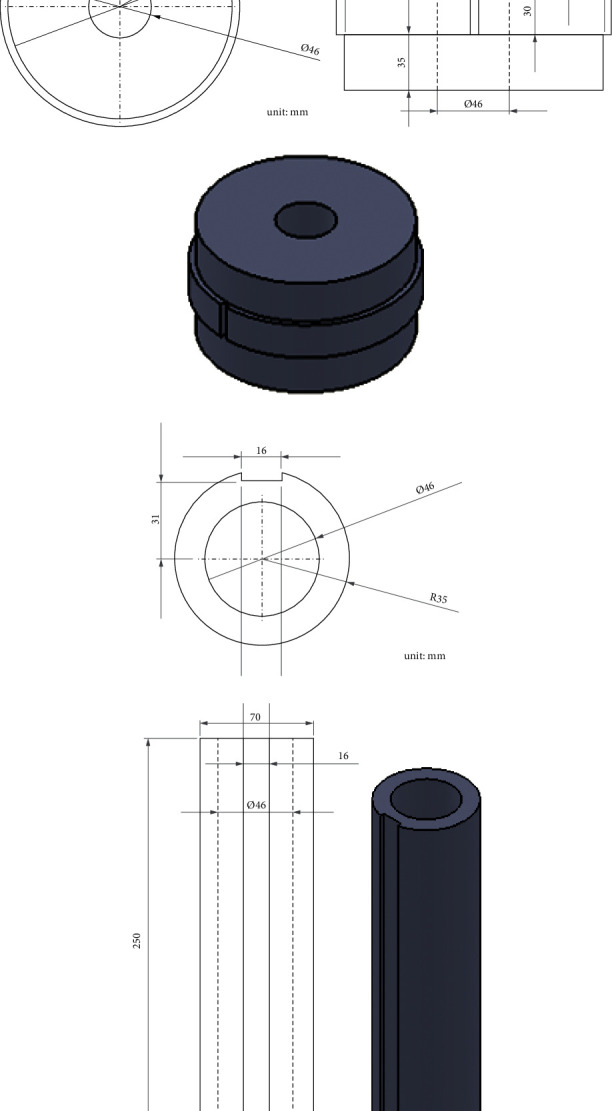
Details about the lead shielding layers of the high- and low-range tubes. (a) Cross section of the lead shielding layer of the high-range tube. (b) Dimensions of the lead shielding layer of the high-range tube. (c) Photo of the physical lead shielding layer of the high-range tube. (d) Cross section of the lead shielding layer of the low-range tube. (e) Dimensions of the lead shielding layer of the low-range tube. (f) Photo of the physical lead shielding layer of the low-range tube.

As mentioned before, the lead shielding layer can be divided into two parts: the lead shielding layer for the high-range tube, and that for the low-range tube. To obtain the angle of the radiation source relative to the finder during dose rate measurement, the lead shielding layer should be thickened in all directions except the preset angle of 0 degrees. The lead shielding layers of the high- and low-range tubes are illustrated in Figure 1. A mold was prepared for the lead shielding layer of each tube. Then, molten lead was poured into the two molds, producing the physical lead shielding layers.

### 3.4. Determination of Radiation Source Direction

In the presence of a gamma radiation source, the direction of the source is theoretically the direction of the maximum gamma radiation monitored by the finder. Since each cylindrical GM tube is installed vertically to the ground, the probe response to gamma radiation should be isotropic in the horizontal direction. Thus, the gamma radiation was measured as the mean of the values in the 360 degrees. So, it is difficult to determine the direction of maximum gamma radiation, solely based on the measured values of gamma radiation.

**Figure 2:**
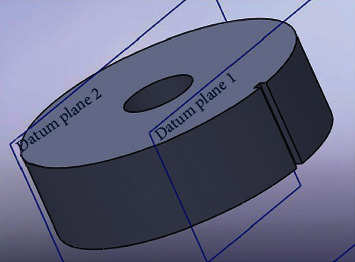
Shape of the lead shielding layer.

In our project, the above problem is solved by specially designing the lead shielding layer for each GM tube, and considering the finder's rotation angle. For the high-range tube, a lead shielding layer was added, with an outer diameter of 176 mm, a thickness of 65 mm, and a height of 30 mm. A hole was opened at the center of the layer, with a diameter of 46 mm. In addition, a groove (width: 5 mm; depth: 5 mm) was cut on the layer to obtain the angle of the tube. For the low-range tube, a lead shielding layer was added, with a thickness of 12 mm, and a height of 250 mm. A hole was opened at the center of the layer, with a diameter of 46 mm. In addition, a groove (width: 5 mm; depth: 4 mm) was cut on the layer to obtain the angle of the tube. The direction of the maximum gamma radiation can be determined based on the finder angle and radiation dose. This is the direction of the gamma radiation source.  Figure 2 shows the shape of the lead shielding layer.

**Figure 3:**
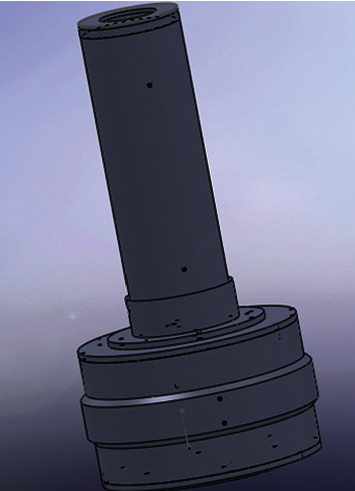
The combined lead shielding layer.

Figure 3 shows the combination of the lead shielding layers for high- and low-range tubes.

**Figure 4:**
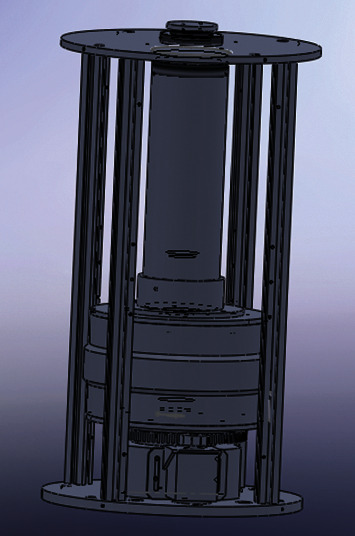
Overall assemblage.

The finder was fixed inside the center hole, whose diameter is 46 mm. After that, the lead shielding layer was fixed on the rotating support platform, such that the finder axis coincides with the platform axis. The platform can rotate 360 degrees, with a step size of 1 degree. By rotating the platform, the angles can be obtained. The overall assemblage is shown in Figure 4.

### 3.5. Estimation of Source-Finder Distance

**Figure 5:**
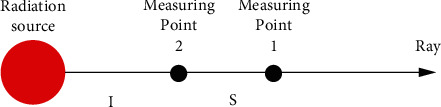
Estimation of source-finder distance.

After the finder detects the direction of maximum gamma radiation, the air kerma rates of two monitoring points in that direction were measured, and the distance between the two points was obtained. Then, the distance from the gamma radiation source and the finder can be estimated by:

(4) $$\begin{array}{c} l=\frac{s}{\sqrt{\left(\overset{•}{ka_{1}}-\overset{•}{ka_{1\text{Background}}}\right)/\left(\overset{•}{ka_{2}}-\overset{•}{ka_{2\text{Background}}}\right)}-1}, \end{array}$$

where *l* is the distance from measuring point 2 to the radiation source (m); *s* is the distance between measuring points 1 and 2 (m); $\overset{•}{ka_{1}}$ and $\overset{•}{ka_{2}}$ are the air kerma rates at measuring points 1 and 2, respectively; $\overset{•}{ka_{1\text{Background}}}$ and $\overset{•}{ka_{2\text{Background}}}$ are the background air kerma rates in the environment of measuring points 1 and 2, respectively.  Figure 5 illustrates how to estimate the source-finder distance.

### 3.6. Neutron Detection

The presence or absence of neutrons was detected with a helium-3 (3He) tube. The tube was encapsulated in the moderator material together with the pre-amplifier circuit. The main-amplifier circuit was also placed in the detector control system for processing.

## 4. Rotating Support Platform

**Figure 6:**
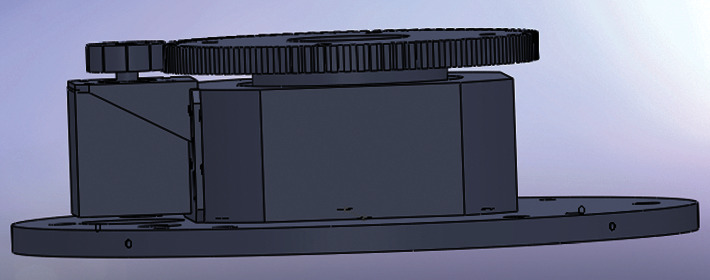
Sketch map of the rotating support platform.

As shown in Figure 6, the rotating support platform is composed of a 1-mode 140-tooth gear driven by a stepping motor. Number of teeth = 360 degrees/step angle *∗* number of beats. The lead shielding layer is fixed on that gear. The stepping motor has a 1-mode 28-tooth gear. Thus, the angular speed ratio between the motor and the gear of the platform is 5 : 1.

There are 1,600 pulses in one rotation of the stepping motor. Therefore, the minimum stepping angle of the lead shielding layer can be 360/(5 *∗* 1600) = 0.045 degrees, which meets the design requirements.  Table 4 lists the technical parameters of the stepping motor.

In light of the final installation effect, the maximum rotation speed of the lead shielding layer was limited to 360 degrees/15 s.

## 5. Detection-Control System

**Figure 7:**
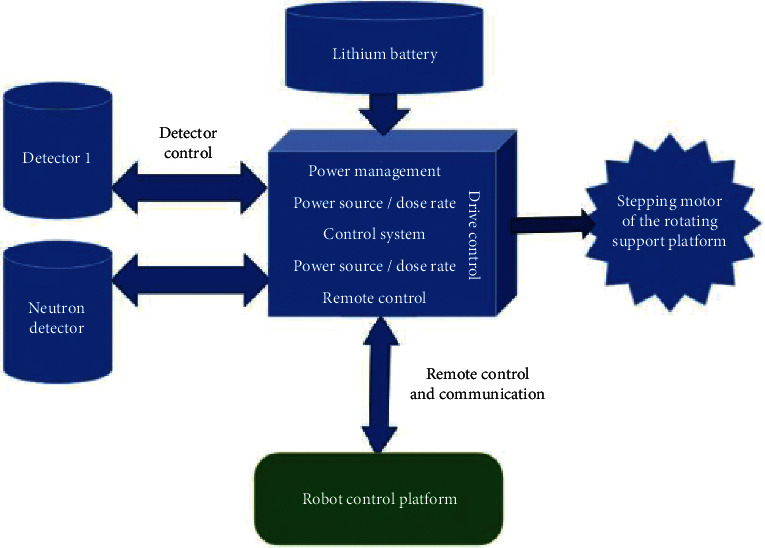
Detection-control system.

As shown in Figure 7, a detection controller was fixed on the robot and wired to the detectors. The controller provides a power of 12 VDC to the detector, which consumes a power of about 50 mA@12 VDC, while receiving the pre-amplifier pulse signal from the detector. The controller also controls the switch of the detector between low and high ranges. Apart from the provision of a power source, the detection controller offers the detector a bias voltage, supports dose rate calculation and logic control, and communicates with the robot control platform.

The controller drives the stepping motor of the rotating support platform with a highly refined step size, such that the platform rotates at an appropriate speed. Meanwhile, the controller collects the dose rate data of the detector and reports the dose rate data and the corresponding angles directly to the console through the robot control platform.

To support the flexible operation of the robot control platform, the detection controller also opens the protocol interface of the cycle setting to the platform. Thus, the measuring cycle can be configured on the robot control platform in the range of 2–3,600 s.

The power source of the detection controller is uniformly provided by the robot control platform. The storage design of the control system meets the requirement on the cumulative dose of 1,560 Gy.

The data packets between the detection controller and the robot control platform are in the format of JASON. The content of the protocol is shown in the attached table. The RS232 interface was selected for the communication between the detection controller and the robot control platform. The detection controller needs a power source of 3 A@12 VDC (two-wire connector): the detection controller and the robot. The detection controller and the robot control platform at least exchange the following information: output dose rate and angle as well as output state. The controller receives the following commands from the robot: forward rotation angle, reverse rotation angle, rotation angle to the target, reset signal, etc.

The detection controller consumes the greatest power (1.3 A@12 VDC) when the motor starts. When the motor is static, the total power consumption of the device is 0.5 A@12 VDC.

## 6. Intelligent Source Detection Algorithm

The automatic search of radiation resources can be decomposed into two tasks: (1) estimating the intensity distribution of spatial radiation and (2) planning the robot trajectory to guide the robot to the position of the maximum radiation intensity.

In this study, the intensity distribution of spatial radiation is estimated as follows: According to the coordinates of some points in the known search area and their radiation intensities, the radiation intensity in the entire search area is estimated. The estimation is a multiple nonlinear regression problem. Many researchers have developed solutions to the problem. The most common solution is a support vector machine (SVM) or artificial neural network (ANN).

The basic idea of the SVM is to define a kernel function, which maps the input vector to a high-dimensional feature space, and construct the optimal separating hyperplane in that space. By choosing a suitable mapping function, most problems with a linearly inseparable input space can be converted into a linearly separable problem in the feature space [7–11].

Based on the backpropagation (BP) learning algorithm, the ANN can solve nonlinearly separable problems. The algorithm trains the network through sample learning. After the training, the network structure represents the mapping from independent variables to dependent variables [9, 12, 13]. In this study, a BP neural network was used to construct a model for the multivariate nonlinear regression problem. The BP neural network has an input layer and an output layer. Therefore, taking the longitude and latitude coordinates as the input of the neural network, the input layer has two neuron nodes; taking the radiation dose as the output of the neural network, the output layer has one neuron. The hidden layer design of the BP neural network includes the determination of the number of hidden layers and the determination of the number of nodes in each hidden layer. The determination of this part needs to be comprehensively considered based on prior experience and the effects of multiple experiments. In terms of determining the number of hidden layers, if the number of hidden layers is too small, the learning ability of the neural network model will be poor, and it is difficult to reflect the mapping relationship between input and output; if the number of hidden layers is too large, the neural network will become complex, resulting in longer training time. Under ideal conditions, the radiation dose of the radiation field is inversely proportional to the square of the distance. In the actual process, it is very complicated by the superposition of fields, the influence of the atmosphere and the Earth and other factors.

Nevertheless, both SVM and BP algorithm have some defects. The SVM calls for the design of very complex parameters. As for the BP algorithm, the number of layers and the number of nodes on each layer must be determined for the network. If these parameters are not designed properly, a high error will occur. Both approaches are rather complex.

This study chooses Gaussian process regression (GPR) to estimate the intensity distribution of spatial radiation, and plan the robot trajectory, because the GPR boasts two strengths: First, the GPR can output the predicted values and their distribution, i.e., the mean and variance of Gaussian distribution. The predicted values represent the radiation intensity, and the distribution of predicted values reflects the uncertainty of radiation intensity. Second, the GPR algorithm is simple to realize. During the calculation, it is only necessary to determine the mean function and covariance function of the Gaussian process (GP). The GPR is a nonparametric model that performs the regression analysis on data, using GP priors [14].

**Figure 8:**
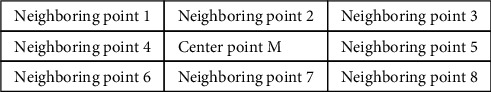
Center point *M* and its neighboring points.

To speed up the search and quickly obtain the measured results, it is necessary to discretize the continuous space. This study discretizes the continuous search area into square grids with a fixed side length. The side length depends on the accuracy required by the system. Each grid was divided into 9 parts, including one center point and eight neighboring points (Figure 8). During the search, the robot records the Cartesian coordinates and detects the radiation intensity at the center point of the grid.

**Figure 9:**
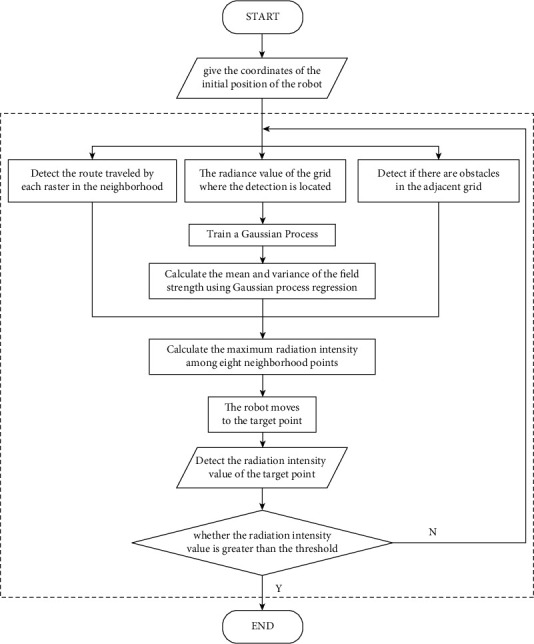
Radioactive source search algorithm flowchart.

Before the search, the initial coordinates *M*(*x*, *y*) of the robot were given. Then, the radiation intensity in the grid of *M* was detected, and saved in a one-dimensional (1D) array *E*, yielding the training set [*X*, *E*]. The training set was adopted to train the selected GP. After the training, the GPR was employed to solve the mean and variance of the radiation intensity of the 8 neighboring points. In this way, the maximum radiation intensity among the 8 neighboring points was identified. The neighboring point with the maximum value was called the target neighboring point. Finally, the robot moved to the target neighboring point and to detect the radiation intensity at that point. If the detected intensity is greater than the threshold, the search would be terminated. Otherwise, a new round of GPR would begin at the coordinates of the target neighboring point. The flow chart of the specific algorithm is shown in Figure 9.

## 7. Conclusions

Since the nascence of nuclear weapons and the nuclear industry, radiation sources have gone out of control and lost occasionally. So far, the uncontrolled radiation sources have been searched for manually. The manual search greatly threatens the health of the searchers. Thus, it is of far-reaching influence to develop a search robot for uncontrolled radiation sources. Contraposing the search of uncontrolled radiation sources, this study designs a detection system for a radiation source search robot. In the subsequent research, the research team will devise a reliable robot source search algorithm and verify the effectiveness of the algorithm through computer simulation and physical simulation.

## Figures and Tables

**Table 1:** Performance indices of high/low range GM tubes.

Serial number	Performance	Low range	High range
1	Model	70031	70018
2	Material	Energy compensation GM tube	Energy compensation GM tube
3	Dose rate range	0.1 *μ*Gy/h–3 mGy/h	0.1 m Sv/h–20 Sv/h
4	Energy range	35 keV–1.3 MeV	70 keV–4 MeV
5	Sensitivity (662 keV, 137 Cs)	16 count/s/*μ*Gy/h	16 counts/s/mSv/h
6	Life	6 × 10^10^ pulses	6 × 10^10^ pulses
7	Working environment	−40°C–70°C	−50°C–70°C
8	Probe size	Φ32 *∗* 300 mm	Φ40 *∗* 90 mm

**Table 2:** The conversion factor from *Ka* to *H* *∗* (10).

Photon energy	*H* *∗* (10)/*Ka*	Photon energy	*H* *∗* (10)/*Ka*
MeV	Sv/Gy	MeV	Sv/Gy
0.015	0.26	0.500	1.23
0.020	0.61	0.600	1.21
0.030	1.10	0.800	1.19
0.040	1.47	1	1.17
0.050	1.67	1.5	1.15
0.060	1.74	2	1.14
0.080	1.72	3	1.13
0.100	1.65	4	1.12
0.150	1.49	5	1.11
0.200	1.40	6	1.11
0.300	1.31	8	1.11
0.400	1.26	10	1.10

**Table 3:** Performance indices of neutron probe.

Serial number	Performance	
1	Energy range	0.025 eV–14 MeV
2	Sensitivity	0.5 CPS/(*μ*Sv/h), 252 Cf source
3	Life	6 × 10^10^ pulses
4	Working environment	Temperature: −10°C–50°C; humidity ≤ 90% (30°C, non-condensing)
5	Warning method	The light-emitting diode (LED) light flickers, along with an alarm sound.
6	Probe size	Φ32 *∗* 300 mm
7	Weight	600 g

**Table 4:** Technical parameters of the stepping motor.

Stepping angle	Static torque	Current	Axial length	Axial diameter	Weight
1.8	0.5	0.17	16	8	0.37

## Data Availability

The data used to support the findings of this study are available from the corresponding author upon request.
